# An assessment of direct and indirect costs of dementia in Brazil

**DOI:** 10.1371/journal.pone.0193209

**Published:** 2018-03-01

**Authors:** Ceres Ferretti, Flávia M. Sarti, Ricardo Nitrini, Fernando F. Ferreira, Sonia M. D. Brucki

**Affiliations:** 1 Department of Neurology, Cognitive and Behavioral Neurology Unit, School of Medicine, Universidade de São Paulo, SP—Brazil; 2 Department of Nursing and Nutrition of Universidade de Taubaté, Taubaté—São Paulo Brazil; 3 Universidade de São Paulo, USP Leste, Escola de Artes, Ciências e Humanidades, São Paulo, SP—Brasil; 4 Hospital Santa Marcelina, São Paulo, SP—Brasil; Boston University School of Public Health, UNITED STATES

## Abstract

**Background:**

To analyze costs associated with dementia based on a cross-sectional study in the Brazilian health system.

**Methods:**

Direct and indirect costs were estimated by conducting comprehensive interviews on the use of resources in a sample of 156 patients with dementia treated at an outpatient memory clinic of a tertiary hospital. A regression model was used to determine the main determinants of costs associated with dementia.

**Results:**

Global costs of dementia were US$1,012.35; US$1,683.18 and US$1,372.30 per patient/month for mild, moderate and severe stages, respectively. Indirect costs ranged from US$536.62 to US$545.17 according to severity. Dementia costs were influenced by medication, FAST score, and educational level of caregiver.

**Discussion:**

The study represents an original contribution toward establishing direct and indirect costs of dementia in Brazil. Results indicate significant economic impacts, including projection of annual costs of US$16,548.24 per patient.

## Introduction

Recent estimates of the prevalence of dementia and Alzheimer’s disease (AD) have risen dramatically around the world, increasing from 47.47 million individuals in 2015 to a predicted 75.63 million in 2030 and 135.46 million in 2050 [1]. In Latin America (LA) as a whole, the number of patients with dementia was approximately 3 million in 2010, with Brazil accounting for over 1 million cases [2,3]. Evidence shows variations in prevalence according to region of the country, although 12% is the currently accepted rate [4–6].

Recent studies indicate escalating costs for treatment of dementia rising from US$604 billion in 2010 to US$818 billion in 2015 [7–10]. With regard to projections of demographic transition, particularly among upper-middle-income countries (UMICs), the financial burden associated with the emergence of non-communicable diseases (NCDs) associated with human aging may have a substantial economic impact within public national health systems.

Brazil is the largest country in LA, with a population of approximately 207 million, comprising 24 million elderly individuals. Major changes in terms of life expectancy and population dependency ratio have occurred in Brazil over the last few decades, posing a challenge to the national health system, which is predominantly based on public funding associated with universal health care coverage [11–13].

The onset of dementia compromises patient quality of life, family relationships, and productivity of patients’ relatives and caregivers. Based on epidemiological data [1,2], governments worldwide are discussing the need for health policies related to dementia, to ensure provision of adequate care and support to patients diagnosed with dementia and to their respective caregivers [10,14–16].

Assessments of the economic impact of diseases on national health systems yield evidence to inform public policies on health, allowing the improvement of health strategies based on a “societal” perspective. Regarding Alzheimer’s disease, there are few studies discussing the costs of dementia in LA, whether direct, indirect or societal costs, due to methodological difficulties involving monetary valuation methods and cost-effectiveness analysis [13,15].

The present study represents an original contribution toward establishing the direct and indirect costs of dementia in Brazil, adopting a methodological framework internationally recommended by experts in the field of knowledge [17,18], using societal perspective to assess the burden of the disease on patients and caregivers. The results reported are drawn from the first phase (cross-sectional stage) of a longitudinal investigation entailing the description of methods and the cost analysis associated with dementia within the Brazilian health system (CAAD project) [18].

## Methods

### Sample selection

A sample of 156 patients attending outpatient health care at the Neurology and Cognitive Behavior Unit of Hospital das Clinicas (Sao Paulo, Brazil) was selected after individual assessment by a neurologist. Comprehensive interviews were performed between November 2011 and May 2015.

The inclusion criteria for patient participation in the study were having a diagnosis of dementia at mild, moderate or severe stages with formal or informal caregivers who cared for the patient. The exclusion criteria were patients diagnosed with Mild Cognitive Impairment (MCI).

The statistical precision for estimation of direct and indirect costs of dementia was based on hypothesis test to calculate sample size and infer expected values of the target variable: considering one single sample, the idea was to estimate the distance between true value (population) and the sample value by controlling the statistical significance and power. According to Chow et al. [19] and Chow and Liu [20], the sample size may be calculated from the following hypotheses:

$$H_{0}:\;|\bar{x}-\mu_{0}|\geq \delta \;versus\;H_{a}:\;|\bar{x}-\mu_{0}|<\delta ,$$

Where $\bar{x}-\mu_{0}$ is the meaningful difference between the population mean μ_*o*_ and the observed value $\bar{x}$ (null hypothesis). The constant δ is the statistical precision measuring the distance between the real value and the value obtained from sample of size *n*_*s*_.

The study was based on calculation of the sample size taking into account both the Type I error (α) and the Type II error (β). In this case, it is important to specify the statistical power (1 - β) 100% and the confidence level (1- α) 100%. [19] derived the following expression to the sample size *n*_*s*_:

$$n_{s}\geq \frac{{\left(t_{\alpha ,n_{s}-1}+t_{\frac{\beta }{2},n_{s}-1}\right)}^{2}}{\theta^{2}}$$ [Eq 1]

Where $\theta =\frac{\delta }{s_{N}}$. We denote by *S* the sample standard deviation and $s_{N}^{2}=S^{2}\frac{N-n_{s}}{N-1}$ is the corrected variance. The correction factor $pcf=\sqrt{\frac{N-n_{s}}{N-1}}$ is applied for finite population of size *N*, which is the present case. We assumed that the variance is unknown. The scores $t_{\alpha ,n_{s}-1}$ and $t_{\beta /2,n_{s}-1}$ are calculated from a t-distribution with (*n*_*s*_-1) degrees of freedom.

The average value for treatment cost found from our sample was $\bar{x}$ = $1,405.72; the standard deviation *S* = $1,376.28; corrected standard deviation *S*_N_ = $1,300.04; and the population *N* = 1,440.

Using Eq 1, we estimated *n*_*s*_ by controlling θ, the significance level α and statistical power 1-β simultaneously. Setting α = 5% and 1-β = 80% and θ = 0,2; the sample size *n*_*s*_ = 156. That means the precision of estimates is δ~$260.00; which leads to the conclusion that the population costs should not exceed $\bar{x}$ by the amount of δ~$260.00; i.e., $\left|\bar{x}-\mu_{0}\right|$<260. Of course, if there is need of higher precision, the sample size must be increased according to Eq 1.

The population size was calculated using information from the Hospital das Clinicas, the largest hospital in Brazil, based on the assessment of 15 elderly patients per week during the period of two years (24 months).

The protocol of the study established that the nurse responsible for diagnosis of dementia among elderly patients in the hospital should invite all caregivers to participate in the study during the period of research. Caregivers who accepted participation in the study were included in the sample, except if patients were diagnosed with Mild Cognitive Impairment (MCI). Thus, every patient diagnosed with mild, moderate or severe dementia and his/hers relatives or caregivers were included in the study.

Patients and caregivers were verbally informed on the details of the project and interviewed only after signing a consent form agreeing to participate in the study. Caregivers responsible for patients’ care signed the consent form, in case of impaired capacity of the patient, being certified that caregivers understood the terms of the research and the rights of participants.

The study was submitted to the Ethics Committee of Hospital das Clinicas of the University of Sao Paulo (HC-FMUSP) and approved under process number 368.096/2013.

### Data collection and processing

Data collection was performed using a research protocol based on semi-structured questionnaire designed to gather information on sociodemographic, economic and health characteristics of patients and caregivers, information on health resources utilization and results of clinical assessment of patients and their respective caregivers, applied by a nurse specialized in dementia.

Clinical data included variables related to disease progression and comorbidities of the patients and their respective caregivers. Detailed information on the research protocol is available within a study published with preliminary results of the research, referring to indirect costs of dementia in Brazil, including a fraction of the planned sample regarding data from caregivers [14].

Disease severity was categorized using the Functional Assessment Staging—FAST scale [21], which allows observation of cognitive impairment due to dementia and dependency stage of the patient, categorizing into seven stages (1 to 7f). In the study, the seven stages were aggregated into three levels (mild, moderate and severe), considering the following scores: “1–4” = mild; “5a-5e” = moderate; and “7a-7f” = severe.

Caregiver burden due to the patient condition was assessed by the ZARIT Burden Interview Scale and categorized into four levels: light to moderate burden (score ranging from 0 to 40), moderate to severe burden (score 41–60) and severe burden (score 61–88) [22].

Information on health resource utilization was based on the Resource Utilization in Dementia (RUD) scale, including variables related to direct costs associated with patient disease and indirect costs related to the relatives’ and caregivers’ job abandonment, early retirement and productivity loss due to burden associated with patient care [23].

Costs estimates were calculated using the information on health resource utilization for direct and indirect costs due to patient condition from a societal perspective. Values were expressed in Brazilian currency (*Reais*), corrected for inflation to February 2016, and converted into U.S. dollars using the official exchange rate released by the Brazilian Central Bank.

Direct costs included costs of patients’ medication, health care, and other resources (excluding medication and health care, e.g., diapers, transportation, and other). Costs on medication prescribed for patients’ treatment were calculated for daily recommended dosage based on patient medical records and caregiver information, using market prices. Costs of health service utilization by patients were estimated using information on health care provided by the Brazilian government within the national health system encompassing specialized inpatient and outpatient health care. In summary, the direct costs included direct private costs (covered by patients’ families) or direct public costs (covered by government funding of national health system).

Indirect costs included costs of caregivers’ health care due to health problems related to burden of patients’ care, costs due to job abandonment or early retirement related to patients’ care and productivity losses of relatives and/or caregivers due to the burden of patients’ condition.

Estimates of economic losses due to job abandonment and early retirement were based on hourly wages according to respective caregiver’s salary, and economic losses due to productivity losses of relatives and/or caregivers time for patients’ care were based on hourly wages per capita, according to reported household income and number of salaried workers reported in the family unit, in order to account for rotation of the task among diverse individuals in the household and to consider differences in wages of household members among formal and informal labor activities.

The calculation of monthly hours related to activities of daily living (ADL), instrumental activities of daily living (IADL) and supervision of the patient was made using previously tested methodology [14].

### Statistical procedures

Data on patients’ and caregivers’ sociodemographic, economic and health characteristics, clinical assessments of patients, and direct and indirect costs due to dementia, according to FAST category, were analyzed by descriptive statistics and partial correlations in order to allow analysis of differences between groups of patients and caregivers, adopting a significance level of 0.05.

Additionally, robust multiple regression models were estimated using the Stata software version 11.2, in order to identify main determinants of dementia costs in the sample of patients in Brazil, adopting 0.05 significance level for inclusion of variables in the models.

## Results

One hundred and fifty-six interview protocols were performed with informal caregivers of outpatients with dementia, categorized according to severity into mild (n = 61), moderate (n = 74) and severe (n = 21) stages of the disease. Five protocols were excluded: one due to missing information and four to duplicate interviews where, in these cases, only data from the latest interview were considered for the data analysis.

Sociodemographic and clinical characteristics of patients indicated that average time of progression of dementia was 60.13 (±41.44) months; and main comorbidities were systemic arterial hypertension (60.26%), followed by cardiovascular disease (40.15%), and diabetes mellitus (27.56%) (Table 1).

**Table 1:** Sociodemographic characteristics and clinical status of patients, according to patient FAST category. Brazil, 2016.

Patient characteristics		Patient dementia stage				
		Mild	Moderate	Severe	Total	Sig.
Patients	N	61	74	21	156	
	%	39.10	47.44	13.46	100.00	
Sex						
Male	(%)	39.34	43.24	42.86	41.67	#
Female	(%)	60.66	56.76	57.14	58.33	#
Age (years)	Mean	72.23	72.97	74.57	72.90	#
	SD	9.99	10.62	9.57	10.20	
Retired	(%)	85.25	79.73	76.19	81.41	#
Income (US$)	Mean	273.04	323.12	195.92	286.41	#
	SD	233.91	444.56	163.60	345.74	
Diagnosis						
Alzheimer's disease	(%)	63.93	74.32	57.14	67.95	#
Vascular dementia	(%)	4.92	0.00	0.00	1.92	#
Frontotemporal dementia	(%)	4.92	2.70	19.05	17.10	#
Lewy Body dementia	(%)	0.00	1.35	0.00	0.64	#
Other	(%)	26.23	21.62	23.81	23.72	#
Disease evolution (months)	Mean	51.48	60.82	82.86	60.13	*
	SD	39.04	39.39	47.99	41.44	
Comorbidities (n)	Mean	2.13	2.12	1.38	2.03	#
	SD	1.36	1.32	1.50	1.38	
Diabetes	(%)	32.79	25.68	19.05	27.56	#
Hypertension	(%)	62.30	67.57	28.57	60.26	*
Cerebrovascular disease	(%)	22.95	17.57	23.81	20.51	#
Cardiovascular disease	(%)	44.26	52.70	28.57	46.15	#
Other	(%)	50.82	45.95	38.10	46.79	#

Note

* p≤0.05

# p>0.05. SD = standard deviation.

Regarding caregiver characteristics, caregivers had predominantly female gender (82.69%), mean age of 54.21 (±14.28) years, and educational level of 9.43 (±5.68) years. Most caregivers lived with the patient, were generally the patient’s sons/daughters, and reported mild or moderate burden of disease, independently of patient dementia stage. Almost 38% of caregivers reported having a diagnosed illness within approximately 12 months of initiating patient care. Main complaints reported were psychological distress and depressive disorders, while 70.51% of caregivers were in use of prescribed medication at the time of interview (Table 2).

**Table 2:** Sociodemographic characteristics, level of caregiver burden and disease after initiating care. Brazil, 2016.

Caregiver characteristics		Patient dementia stage				
		Mild	Moderate	Severe	Total	Sig.
Sex						
Male	(%)	19.67	14.86	19.05	17.31	#
Female	(%)	80.33	85.14	80.95	82.69	#
Age (years)	Mean	55.62	53.11	54.00	54.21	#
	SD	13.43	14.73	15.39	14.28	
Educational level (years)	Mean	8.69	10.22	8.81	9.43	#
	SD	6.46	5.26	4.35	5.68	
Marital status						
Married/Living with partner	(%)	59.02	72.97	66.67	66.67	#
Divorced/Widower/Bachelor	(%)	40.98	27.03	33.33	33.33	#
Number of children	Mean	0.84	0.81	0.67	0.80	#
	SD	1.02	0.81	0.80	0.89	
Salaried work	(%)	40.98	36.49	33.33	37.82	#
Living with patient	(%)	72.13	75.68	66.67	73.08	#
Relationship with patient						
Spouse	(%)	39.34	36.49	23.81	35.90	#
Son/Daughter	(%)	52.46	51.35	52.38	51.92	#
Other	(%)	8.20	12.16	23.81	12.18	#
ZARIT score						
Mild burden	(%)	72.13	36.49	57.14	53.21	*
Moderate burden	(%)	24.59	58.11	19.05	39.74	*
Severe burden	(%)	3.28	5.41	23.81	7.05	*
Disease diagnosed after initiating patient care	(%)	32.79	33.78	66.67	37.82	*
Lag period from diagnosis (months)	Mean	9.03	11.64	18.62	11.56	#

Note

* p≤0.05

# p>0.05. SD = standard deviation.

Utilization of health care resources by patients with dementia and their respective caregivers showed that most medication was provided by the public funded health system (26.28%) or acquired using public and private resources (62.18%). Regarding use of health care resources, statistically significant differences among patients at different severity stages were identified for use of medication, physician visits, and need for professional support of a nurse; whilst among caregivers, no significant differences in utilization of health services were found (Table 3).

**Table 3:** Use of health care resources by patients with dementia and respective caregivers, according to patient FAST category, Brazil, 2016.

Variables		Patient dementia stage				
		Mild	Moderate	Severe	Total	Sig.
Utilization of health system resources for patient care						
Medications prescribed (per day)	Mean	3.43	4.78	2.43	3.94	*
	SD	3.28	4.07	1.80	3.62	
Private purchase only	(%)	13.11	6.76	23.81	11.54	*
Public health system only	(%)	32.79	21.62	23.81	26.28	*
Both public and private	(%)	54.10	71.62	52.38	62.18	*
Hospitalizations (n)	Mean	0.07	0.12	0.10	0.10	#
	SD	0.25	0.37	0.30	0.32	
Inpatient stay (days)	Mean	0.13	0.28	0.10	0.20	#
	SD	0.64	1.38	0.30	1.04	
Emergency room (n)	Mean	0.23	0.24	0.10	0.22	#
	SD	0.62	0.46	0.30	0.51	
Health professional consultations						
Specialized physician (n)	Mean	0.95	0.74	0.95	0.85	#
	SD	1.63	0.68	0.86	1.16	
Physician visits (n)	Mean	0.13	0.50	0.90	0.41	*
	SD	0.39	1.20	2.61	1.29	
Nurse visits (n)	Mean	0.08	0.20	0.67	0.22	#
	SD	0.28	0.89	2.61	1.15	
Professional support						
Nurse	(%)	9.84	18.92	38.10	17.95	*
Household/hospital assistance	(%)	1.64	0.00	0.00	0.64	#
Public/private transportation	(%)	21.31	24.32	9.52	21.15	#
Other	(%)	14.75	29.73	14.29	21.79	#
Utilization of health system resources by caregiver due to caregiving						
Hospitalization (n)	Mean	0.00	0.01	0.00	0.01	#
	SD	0.00	0.12	0.00	0.08	
Inpatient stay (days)	Mean	0.00	0.01	0.00	0.01	#
	SD	0.00	0.12	0.00	0.08	
Emergency room (n)	Mean	0.18	0.38	0.48	0.31	#
	SD	0.47	0.79	1.44	0.81	
Health professional consultations						
Specialized physician (n)	Mean	0.52	0.81	0.43	0.65	#
	SD	0.72	2.03	0.75	1.50	

Note

* p≤0.05

# p>0.05. SD = standard deviation.

Productivity losses of caregivers for patient care were analyzed in relation to total or partial job abandonment data, allowing the estimation of indirect costs of dementia: a total of 373.04 (±251.29) hours dedicated to ADLs, IADLs and supervision of patients, comprising 87.75 hours on ADLs, 129.44 hours on IADLs, and 161.26 hours on supervision (Table 4).

**Table 4:** Characteristics of productivity losses and economic impact for caregivers, according to patient FAST category. Brazil, 2016.

Variables		Patient dementia stage				
		Mild	Moderate	Severe	Total	Sig.
Caregiver job abandonment	(%)	40.98	45.95	47.62	44.23	#
Reasons for job abandonment						
Retirement	(%)	16.39	13.51	4.76	13.46	#
Early retirement	(%)	0.00	1.35	0.00	0.64	#
Dismissal	(%)	1.64	0.00	4.76	1.28	#
Health problems	(%)	8.20	2.70	4.76	5.13	#
Patient care	(%)	11.48	14.86	4.76	12.18	#
Other	(%)	3.28	13.51	28.57	11.54	#
Caregiver working hours lost on patient care	Mean	3.57	3.84	4.95	3.88	#
	SD	11.02	14.94	14.36	13.38	
Estimated time spent on patient support (hours) monthly	Mean	243.28	460.74	440.95	373.04	*
	SD	222.18	222.36	275.93	251.29	
Activities of daily living (hours)	Mean	35.90	115.43	140.81	87.75	*
	SD	64.93	92.48	103.48	94.06	
Instrumental activities of daily living (hours)	Mean	91.15	160.66	130.67	129.44	*
	SD	95.27	104.03	111.73	106.13	
Supervision activities (hours)	Mean	123.11	207.76	186.62	171.81	*
	SD	144.75	168.68	150.25	161.26	

Note

* p≤0.05

# p>0.05. SD = standard deviation.

Global costs attributable to dementia were US$1,405.72 (±1,376.28) per month, comprising US$562.09 (±434.82) direct costs and US$843.63 (±1,172.11) indirect costs (Table 5). Significant part of costs attributable to dementia was indirect costs (60.0%), being mostly due to time spent in patients’ care (89.8% of indirect costs); whilst major part of the direct costs was attributable to other resources for patients’ care (72.6%). That is, there is significant burden to private household expenditures due to dementia, considering that substantial share of direct and indirect of dementia costs (approximately 83%) concentrate on utilization of private resources.

**Table 5:** Monthly direct, indirect and global costs due to dementia and regression model with caregiver burden, according to patient FAST category. Brazil, 2016.

Variables		Patient dementia stage				
		Mild	Moderate	Severe	Total	Sig.
Direct costs due to dementia (US$)						
Cost of patient medication	Mean	65.06	68.42	84.42	69.26	#
	SD	50.70	64.79	95.72	64.75	
Cost of health services for patient treatment	Mean	20.62	18.67	20.84	19.84	#
	SD	27.55	17.31	24.68	22.62	
Cost of other resources for patient care, excluding medication and health services	Mean	27.86	8.27	0.00	15.05	*
	SD	43.62	7.01	0.00	30.16	
**Total Direct Costs**	**Mean**	**53.14**	**62.96**	**78.47**	**61.21**	*
	**SD**	**50.45**	**67.39**	**99.35**	**66.82**	
Indirect costs due to dementia (US$)						
Cost of health services for caregiver treatment	Mean	29.89	59.17	62.10	48.12	#
	SD	55.60	103.03	168.47	100.37	
Costs of productivity loss for patient care	Mean	22.79	51.25	37.02	38.21	#
	SD	56.98	132.05	107.98	105.59	
Costs of time spent on patient support	Mean	525.19	1,013.26	529.64	757.31	*
	SD	580.35	1,552.30	475.49	1,163.57	
**Total Indirect Costs**	**Mean**	**577.87**	**1,123.68**	**628.75**	**843.63**	*
	**SD**	**585.81**	**1,554.53**	**493.21**	**1,172.11**	
Global costs due to dementia (US$)						
**Direct and indirect monthly costs due to dementia**	**Mean**	**1,023.71**	**1,724.01**	**1,393.79**	**1,405.72**	*
	**SD**	**719.01**	**1,780.26**	**860.61**	**1,376.28**	

Note

* p≤0.05

# p>0.05. SD = standard deviation.

The main determinants of costs due to dementia were medication, dementia severity and caregiver educational level (Table 6). Patients’ sociodemographic characteristics, patients’ comorbidities, utilization of health services by patients and/or caregivers and other resources did not represent significant influence on the burden of disease. That is, costs of dementia were independent in relation to other health conditions and other health care costs incurred by patients and caregivers; representing costs specifically linked to burden of dementia in the Brazilian health system, instead of being overestimated due to occurrence of other health problems among the patients in the sample.

**Table 6:** Regression model for global costs due to dementia. Brazil, 2016.

Independent variables	β	SE	95%CI	Sig.
Medications prescribed (per day)	0.35	0.07	0.21–0.48	*
FAST score	4.21	0.27	3.68–4.74	*
Educational level of caregiver	0.27	0.04	0.19–0.35	*
R^2^	0.9138			

Note

* p≤0.05; SE = standard error; CI = confidence interval.

## Discussion

The majority of dementia patients have AD (65%) worldwide [1], where an AD prevalence of 68% was detected in the present study sample recruited from a tertiary outpatient memory clinic in Brazil. Regardless of type of dementia, there are numerous concerns over projections of incidence in UMICs, particularly the ageing process. Life expectancy in Brazil has increased in the last few decades, with future increases in the risk of dementia expected [12].

Considering the lack of evidences on dementia costs in Brazil, a previous study was published presenting preliminary findings of the research project referring to indirect costs of dementia in Brazil [14], including a fraction of the planned sample regarding data from caregivers.

### Characteristics of patients and caregivers

Demographic data on patients and caregivers regarding age and gender proved similar to the findings of other studies conducted in LA [1,12,16] and in high-income countries (HICs) [1,24]. Patients and caregivers interviewed in the present study were younger in comparison to other studies conducted in HICs (72.9 years versus ≥75 years, and 54 years versus 65 years, respectively), and in Brazil (42.1 years) [3,25–28]; however, it was consistent with the previous study performed using subsample of the research [14]. Most patients and caregivers were female, and a higher percentage of retirees was observed among the patients in comparison to other studies [3,14].

The educational level of caregivers in the study was lower in comparison to previous studies [26–29]. Some sociodemographic characteristics were consistent with findings from other studies: married caregivers (66.7%), living with the patient (73.1%), spouses (35.9%) and sons/daughters (51.9%), reflecting cultural aspects of the duty to provide elderly with care seen in LA [14], as well as Asian and Eastern countries [27].

A high proportion of caregivers reported leaving formal jobs to take care of patients, and changes in type of formal/informal work were also observed among caregivers with patients at the mild stage (41% versus 11% in other studies [3], including 36% in the preliminary results of the study [14]). Caregivers supporting patients at the severe dementia stage in the sample worked 4.95 fewer hours per month (formal or informal), providing more than one hour of care above the average for the sample.

### Comorbidities

A meta-analysis involving 13,978 elderly individuals in the Brazilian population showed a 68% prevalence of high blood pressure (HBP), a similar rate to the present study in which HBP was the most prevalent comorbidity [29]. Diabetes mellitus (DM) has 6.2% prevalence in Brazil’s adult population and ranges from 14.5% to 19.6%, depending on age group [30]; whilst in the sample analyzed the proportion of patients with DM was significantly higher (27.6%).

Considering evidence on average survival rates after diagnosis (48–96 months) [31,32], the evolution of dementia reported among patients in the sample (60.1 months) is consistent with other studies, including the preliminary results reported in previous study [14].

The long-term evolution of AD may lead to the occurrence of acute conditions coexisting with chronic diseases or, rarely, with dementia only, exposing patients to situations of advanced frailty [33,34]; resulting in additional hidden costs which cause collateral burden to society [35,36].

A recent study involving a sample of 68,844 elderly individuals indicated that patients with dementia had, on average, 3.69 comorbidities versus 2.44 comorbidities among individuals without dementia [25].

The present study found an average of 2.03 (±1.38) comorbidities and a lower prevalence of DM (27.6% vs. 36.1%) and HBP (60.3% vs. 83.5%) compared to the cited study. By contrast, there was a higher prevalence of cardiovascular disease (46.2% vs. 41.2%), and cerebrovascular disease (20.5% vs. 11.3%) [25].

The lower rates of HBP and DM may be due to actions in primary care [25,37]; although the significant prevalence of DM indicates the need for public policies to reduce and/or control complications (including cardiovascular and cerebrovascular diseases), thus avoiding overload of the health system at secondary and tertiary levels [25,27,29,31].

### Caregiver overload

Psychological overload of caregivers in attending dementia patients, economic impacts of the disease, and insidious evolution of AD may be factors contributing to excessive burden among caregivers [14,38]. The time lag between the initiation of patient care and the onset of caregiver illness was 11.56 (±20.90) months, showing that one year of care may be sufficient to cause health problems in caregivers of dementia patients.

Caregivers reported mostly emotional overload and depression; conditions associated with low individual resilience capacity, related to the need to change work hours or to abandonment of profession, leisure activities and ongoing life projects [39–41]. Usual work capacity was preserved for most caregivers of productive age; however, a significant proportion of caregivers reported the need to abandon formal work due to the sick relative.

A pioneering study evaluating the presence of comorbidities among caregivers of dementia patients in Brazil compared to individuals from the general population, indicated higher prevalence of depression (23% versus 11%), major depression (29% versus 20%), decline in physical health (7.3% versus 5.5%), obesity (23% versus 18%), and HBP (23% versus 15%) in caregivers than in non-caregivers, respectively [28]. In the present study, there was higher prevalence of depression (66.7%) than that found by Laks et al. [28]; however, the results were based on self-reported information from the caregiver about diseases diagnosed and on assessment of caregiver burden using a psychometric instrument.

Informal caregivers were more likely to report health problems; information that should be analyzed prospectively by general practitioners, especially considering the need for disease prevention. The risk of onset of physical or mental illness among caregivers may be elevated for numerous reasons ranging from a lack of resources for appropriate caregiving to individual predisposition associated with previous history of life and feeling of loneliness due to the responsibility of caregiving [32,36,40].

### Informal care

Estimation of time dedicated to care of dementia patients and its economic evaluation may be subject to controversies over the methods employed for assessing hours (including ADL, IADL, supervision, or all) and appraisal of hourly costs, including issues related to country economic classification [8,9,14].

This study was based on measurement of global number of hours per month dedicated to ADLs, IADLs and supervision of dementia patients, which totaled 373.04 hours per month as reported by caregivers, 23.5% higher than the average in North America (87.60 hours per month) [3]; however, lower in comparison to the preliminary results (438.37 hours per month) of the research previously reported [14].

The annual time spent by Brazilian caregivers in the sample (4,476.5 hours per year) represents 393% of the average time reported in studies performed in USA (1,139.0 hours per year) [1,3], and approximately double the daily hours (12.4 hours per day), which may explain the overload perceived by caregivers.

### Direct and indirect costs of dementia

A recent 2015 study based on a top-down approach to estimate costs of dementia reported that a significant percentage (87.4%) of costs worldwide were attributable to high-income countries (HICs), comprising predominantly social direct costs (US$308.1 billion, 43.1%) and informal care costs (US$271.1billion, 37.9%), whilst direct medical costs (US$136.0 billion, 19.0%) accounted for a lower proportion [8]. Informal care of dementia patients is usual in LA, due to low family income and high costs of hiring formal caregivers [14,41,42].

In Brazil, the Ministry of Health provides without charge medication for treatment of dementia, hospitalization, specialist consultations and outpatient follow-up for patients and caregivers [43–45]; the present study is pioneer in estimating direct social costs of health care supported by government funding within the Brazilian health system.

Although estimation of costs using the top-down approach (macro-costing technique) may not be as good as estimates based on the bottom-up approach (micro-costing technique) used in the study, the results showed that the economic impact of health care provided by the Brazilian government (direct medical costs) is equivalent to approximately 24% of household income per capita at the mild stage, 21% at the moderate stage, and 43% at the severe stage; representing a significant reduction in health expenditure for dementia patients and their respective families, in contrast to conditions prevailing in other upper-middle income countries (UMICs) [46–48].

Direct costs of dementia reported in the study accounted for US$562.09 (40.0% of global dementia costs). A substantial proportion of dementia costs estimated in the context of the study were attributable to indirect costs; i.e., social costs associated with caregiver productivity loss and time spent by caregiver on patient support. It is important to highlight that total indirect costs per month reported in the study (US$843.63) were higher than family income per capita reported (US$286.41); representing losses of income of US$577.87 for patients at mild stage, US$1,123.68 at moderate stage, and US$628.75 for patients at severe stage.

In comparison to previous study based on preliminary data referring to indirect costs of caregivers [14], the present study showed higher costs due to time spent in patients’ support and productivity losses due to job abandonment (considering solely cases attributable to patients’ care); that is, the partial sample initially interviewed in the research probably present underestimation of indirect costs of dementia in the Brazilian health system.

Based on estimates provided for UMICs [8], global costs of dementia represent approximately US$5,284 per person, of which 57.1% represents informal care costs; 22.4% direct medical costs; and 20.5% social costs. The study indicated that, in Brazil, global costs of dementia are significantly lower (US$1,405.72 per person), with 56.6% attributable to informal care costs; 15.1% direct medical costs; and remaining 37.3% social costs.

Additionally, according to the regression model estimated, the progression of dementia (FAST score) had significant influence on economic impact of the disease, followed by use of medication and educational level of the caregiver. The last two variables represent increased costs associated with direct utilization of resources of the health system and the patient’s family, respectively; whilst the first variable is probably associated with the social burden of dementia, since FAST score showed a significant positive association with ZARIT score on the analysis performed.

The results obtained also highlight the need to improve the quality of basic health care, especially regarding dissemination of information to the population on protective factors to delay the onset of dementia [48,49]. Simple, low-cost interventions may be effective strategies for UMICs, resulting in policies with a positive impact on aging which is inversely associated with exposure to risk of dementia [47].

Global costs of dementia represent a substantial economic and social burden, and comparisons with the impact caused by other diseases may encourage discussion on societal onus, toward prioritizing long-term health strategies to reduce health care costs through disease prevention and health promotion, thereby minimizing the risks of deterioration in the quality of health care [10,33].

The study showed that the global costs of dementia in Brazil already outstrip available resources within Brazilian society, calling for the development of health care strategies based on education programs on dementia (especially in primary care) and training of health professionals to provide caregivers with information, constituting useful approaches to attenuate the current and future impact of dementia in UMICs over the coming decades [14,35,42,47,50].

### Limitations

The main limitation of the study was the utilization of a cross-sectional study design with convenience sampling; given that longitudinal analysis would provide additional evidence on determinants of health care costs and progression of the disease, while random sampling could provide information to perform further extrapolation in the statistical analysis from a societal perspective.

However, considering that the data presented relates to the first stage of the CAAD project, the research group is expected to be able to carry out further economic analysis based on longitudinal data.

Thus, in view of the dearth of evidence on dementia costs in Brazil, it was considered important to disseminate these initial findings on the economic impact of dementia, including direct and indirect costs, in the context of a publicly financed health system with universal coverage.

## Supporting information

S1 Table (DOCX)

## References

[1] World Health Organization (WHO). First WHO Ministerial Conference on Global Action against Dementia: Meeting Report. Geneva: World Health Organization; 2015.

[2] Alzheimer’s Disease International / Bupa. Relatório ADI/Bupa—Demência nas Américas: custo atual e futuro e prevalência da doença de Alzheimer e outras demências. ADI/Bupa; outubro de 2013. Available on: https://www.alz.co.uk/sites/default/files/pdfs/dementia-in-the-americas-BRAZILIAN-PORTUGUESE.pdf [Access Jan 15, 2017].

[3] Alzheimer’s Association. 2016 Alzheimer’s disease facts and figures. Alzheimer’s & Dementia 2016; 12(4). Available on: http://www.alz.org/documents_custom/2016-facts-and-figures.pdf [Access Jan 15, 2017]. 10.1016/j.jalz.2016.03.001 27570871

[4] Cesar KG, Brucki SMD, Takada LT, Oliveira MO, Porto FHG, Senaha MLH, et al Prevalence of cognitive impairment in Tremembe, Brazil. Journal of The Neurological Science 2015; 357(Suppl.1):e123 doi: 10.1016/j.jns.2015.08.396

[5] Brucki SMD, Nitrini R. Cognitive impairment in individuals with low educational level and homogeneous sociocultural background. Dement Neuropsychol 2014; 8(4):345–350. doi: 10.1590/S1980-57642014DN84000007 29213924 10.1590/S1980-57642014DN84000007 PMC5619182

[6] Lopes MA1, Ferrioli E, Nakano EY, Litvoc J, Bottino CM. High prevalence of dementia in a community-based survey of older people from Brazil: association with intellectual activity rather than education. J Alzheimers Dis. 2012; 32(2):307–316. doi: 10.3233/JAD-2012-120847 22785401 10.3233/JAD-2012-120847

[7] World Bank Country and Lending Groups—Country Classification 2017. Available on: http://datahelpdesk.worldbank.org/knowledgebase/articles/906519-world-bank-country-and-lending-groups [Access Jan 17, 2017].

[8] Wimo A, Guerchet M, Ali GC, Wu YT, Prina AM, Winblad B, et al The worldwilde costs of dementia 2015 and comparisons with 2010. Alzheimers Dement. 2017;13(1):1–7. doi: 10.1016/j.jalz.2016.07.150 27583652 10.1016/j.jalz.2016.07.150 PMC5232417

[9] Prince M, Wimo A, Guerchet M, Ali GC, Wu YT, Prina M. World Alzheimer Report 2015: The global impact of dementia: an analysis of prevalence incidence, cost and trends. London: Alzheimer’s Disease International; 2015.

[10] Knut E, Laks J. Towards a Brazilian dementia plan? Lessons to be learned from Europe. Dement Neuropsychol 2016; 10(2):74–78. doi: 10.1590/S1980-5764-2016DN1002002 29213437 10.1590/S1980-5764-2016DN1002002 PMC5642397

[11] Veras RP, Caldas CP, Dantas SB, Sancho LG, Sicsú B, Motta LB, et al Family care for demented elderly individuals: cost analysis. Rev. Psiq. Clín. 2007; 34(1):5–12.

[12] Burlá C, Camarano AA, Kanso S, Fernandes D, Nunes R. Panorama prospectivo das demências no Brasil: um enfoque demográfico. Cien Saude Colet. 2013; 18(10):2949–2956. doi: 10.1590/S1413-81232013001000019 24061021 10.1590/s1413-81232013001000019

[13] Liu Z. Economic costs of dementia in low and middle income countries. London: King’s College; 2012.

[14] Ferretti CEL, Nitrini R, Brucki SMD. Indirect costs with dementia: a Brazilian study. Dement Neuropsychol 2015; 9(1):42–50. doi: 10.1590/S1980-57642015DN91000007 29213940 10.1590/S1980-57642015DN91000007 PMC5618990

[15] Allegri RF, Butman J, Arizaga RL, Machnicki G, Serrano C, Taragano FE, et al Economic impact of dementia in developing countries: an evaluation of costs of Alzheimer-type dementia in Argentina. Int Psychogeriatr 2007;19(4):705–718. doi: 10.1017/S1041610206003784 16870037 10.1017/S1041610206003784

[16] Wimo A Health economic aspects of dementia: Cost of dementia. Luxembourg: Alzheimer Office; 2009. Available on: http://www.alzheimer-europe.org/Research/European-Collaboration-on-Dementia/Cost-of-dementia/Health-economic-aspects-of-dementia [Access Jan 15, 2017].

[17] Winblad B, Amouyel P, Andrieu S, Ballard C, Brayne C, Brodaty H, et al Defeating Alzheimer’s disease and other dementias: a priority for European science and society. The Lancet Neurology 2016; 15(5):455–532. doi: 10.1016/S1474-4422(16)00062-4 26987701 10.1016/S1474-4422(16)00062-4

[18] Ferretti CEL, Nitrini R, Brucki SMD. Description of methods and analyses of cost with dementia. The CAAD project (in course) conducted by Neurology Dept—Cognitive Behavior Unit of Hospital das Clinicas of University of Sao Paulo Sao Paulo, Brazil; 2013.

[19] Chow S-C, Wang H, Shao J. Sample size calculations in clinical research. CRC Press, 2007.

[20] Chow S-C, Liu J-P. Design and analysis of bioavailability and bioequivalence studies. CRC Press, 2008.

[21] Reisberg B, Sclan SG. Functional Assessment Staging (FAST) in Alzheimer’s disease: reliability, validity, and ordinality. International Psychogeriatrics 1992; 4(3):55–69. 1504288 10.1017/s1041610292001157

[22] Scazufca M. Brazilian version of the Burden Interview scale for the assessment of burden of care in carers of people with mental illness. Versão brasileira da escala de Burden Interview para avaliação de sobrecarga em cuidadores de indivíduos com doenças mentais. Rev Bras Psiquiatr 2002; 24(1):12–17. doi: 10.1590/S1516-44462002000100006.

[23] Wimo A, Gustavsson A, Jönsson L, Winblad B, Hsu MA, Gannon B. Application of resource utilization in dementia (RUD) instrument in a global setting. Alzheimer’s & Dementia 2013; 9(4):429–435. doi: 10.1016/j.jalz.2012.06.008 10.1016/j.jalz.2012.06.008 23142433

[24] Wimo A, Prince M. World Alzheimer Report 2010: the global economic impact of dementia. London: ADI; 2010.

[25] Poblador-Plou B, Calderón-Larrañaga A, Marta-Moreno J, Hancco-Saavedra J, Sicras-Mainar A, Soljak M, et al Comorbidity of dementia: a cross-sectional study of primary care older patients. BMC Psychiatry 2014; 14:84 doi: 10.1186/1471-244X-14-84 24645776 10.1186/1471-244X-14-84 PMC3994526

[26] Ferri CP. Population ageing in Latin America: dementia and related disorders. Rev Bras Psiquiatr. 2012; 34(4):371–374. doi: 10.1016/j.rbp.2012.08.005 23429806 10.1016/j.rbp.2012.08.005

[27] Albanese E, Liu Z, Acosta D, Guerra M, Huang Y, Jacob KS, et al Equity in the delivery of community healthcare to older people: findings from 10/66 Dementia Research Group cross-sectional surveys in Latin America, China, India and Nigeria. BMC Health Services Research 2011; 11:153 doi: 10.1186/1472-6963-11-153 21711546 10.1186/1472-6963-11-153 PMC3146820

[28] Laks J, Goren A, Dueñas H, Novick D, Kahle-Wrobleski K. Caregiving for patients with Alzheimer’s disease or dementia and its association with psychiatric and clinical comorbidities and other health outcomes in Brazil. Int J Geriatr Psychiatry 2015; 31(2):176–185. doi: 10.1002/gps.4309 26011093 10.1002/gps.4309

[29] Malachias MVB, Souza WKSB, Plavnik FL, Rodrigues CIS, Brandão AA, Neves MFT, et al 7a Diretriz Brasileira de Hipertensão Arterial. Arq Bras Cardiol. 2016; 107(3 Suppl.3):1–83. ISSN-0066-782X.

[30] Instituto Brasileiro de Geografia e Estatística (IBGE). Pesquisa Nacional de Saúde 2013. Percepção do estado de saúde, estilos de vida e doenças crônicas. Rio de Janeiro: IBGE; 2014. ISBN 978-85-240-4334-5

[31] Xie J. Brayne C, Mathews FE. Survival times in people with dementia: analysis from a population based cohort study with 14-years follow-up. BMJ 2008; 336(7638):258–262. doi: 10.1136/bmj.39433.616678.25 18187696 10.1136/bmj.39433.616678.25 PMC2223023

[32] Brookmeyer R, Corrada MM, Curriero FC, Kawas C. Survival following a diagnosis of Alzheimer disease. Arch Neurol. 2002; 59(11):1764–1767. doi: 10.1001/archneur.59.11.1764 12433264 10.1001/archneur.59.11.1764

[33] Nery AL, Yassuda MS, Araujo LF, Eulálio MC, Cabral BE, Siqueira MEC, et al Methodology and social, demographic, cognitive, and frailty profiles of community-dwelling elderly from seven Brazilian cities: the FIBRA Study. Cad Saúde Pública 2013; 29(4):778–792. doi: 10.1590/S0102-311X2013000400015 23568307

[34] Serra-Prat M, Papiol M, Vico J, Palomera E, Sist X, Cabré M. Factors associated with frailty in community-dweling elderly population. A cross-sectional study. European Geriatric Medicine 2016; 7(6):531–537. doi: 10.1016/j.eurger.2016.09.005

[35] Wimo A, Ballard C, Brayne C, Gauthier S, Handels R, Jones RW, et al Health economic evaluation of treatments for Alzheimer’s disease: impact of new diagnostic criteria. Journal of Internal Medicine 2014; 275(3):304–316. doi: 10.1111/joim.12167 24605810 10.1111/joim.12167

[36] Meyer HJC, Harirari P, Schellack N. Overview of AD and its management. S Afr Pharm J. 2016; 83(9):48–56.

[37] Facchini LA, Piccini RX, Tomasi E, Thumé E, Silveira DS, Siqueira FV, et al Performance of the PSF in the Brazilian South and Northeast: institutional and epidemiological Assessment of Primary Health Care. Ciência & Saúde Coletiva 2006; 11(3):669–681. doi: 10.1590/S0104-80232006000200014

[38] Pinquart M, Sorensen S. Associations of stressors and uplifts of caregiving with caregiver burden and depressive mood: a meta-analysis. J Gerontol B Psychol Sci Soc Sci. 2003; 58(2):112–128. 10.1093/geronb/58.2.p112 12646594

[39] Zarit S. Positive aspects of caregiving: more than looking on the bright side. Aging Ment Health 2012; 16(6):673–674. doi: 10.1080/13607863.2012.692768 22746192 10.1080/13607863.2012.692768

[40] Mausbach BT, Chautillion EA, Roepke SK, Patterson TL, Grant I. A comparison of psychosocial outcomes in elderly Alzheimer caregivers and noncaregivers. Am J Geriatr Psychiatry. 2013; 21(1):5–13. doi: 10.1016/j.jagp.2012.10.001 23290198 10.1016/j.jagp.2012.10.001 PMC3376679

[41] Prince M, Ferri CP, Acosta D, Albanese E, Arizaga R, Dewey M, et al The protocols for the 10/66 dementia research group population- based research programme. BMC Public Health 2007, 7:165 doi: 10.1186/1471-2458-7-165 17659078 10.1186/1471-2458-7-165 PMC1965476

[42] Mendes EV. 25 anos do Sistema Único de Saúde: resultados e desafios. Estudos Avançados 2013; 27(78): 27–34. ISSN 0103–4014

[43] Brandão CMR, Guerra Junior AA, Cherchiglia ML, Andrade EIG, Almeida AM, Silva GD, et al Gastos do Ministério da Saúde do Brasil com medicamentos de alto custo: uma análise centrada no paciente. Value in Health 2011; 14(Suppl.5):S71–S77. 21839903 10.1016/j.jval.2011.05.028

[44] Gutierrez BAO, Silva HS, Guimarães C, Campino AC. Economic impact of Alzheimer’s disease in Brazil: is it possible to improve care and minimize costs? Ciência & Saúde Coletiva 2014, 19(11):4479–4486. doi: 10.1590/1413-812320141911.03562013. 25351314 10.1590/1413-812320141911.03562013

[45] Xu J, Wang J, Wimo A, Fratiglioni L, Qiu C. The economic burden of dementia in China, 1990–2030 Implications for the health policy. Bulletin of World Health Organization 2017; 95(1): 18–26. ISSN 1564060400429686 10.2471/BLT.15.167726 PMC5180346 28053361

[46] Parkinson M, Carr SM, Mushmer R, Abley C. Investigating what works to support family carers of people with dementia: a rapid realist review. J Public Health 2016; (Epub ahead of print):1–12 27679663 10.1093/pubmed/fdw100 PMC5939885

[47] Dekosky ST, Gandy S. Environmental exposures and the risk for Alzheimer’s disease: can we identify the smoking guns? JAMA Neurol. 2014; 71(3):273–275. doi: 10.1001/jamaneurol.2013.6031 24473699 10.1001/jamaneurol.2013.6031 PMC4847540

[48] Buchman AS, Boyle PA, Yu L, Shah RC, Wilson RS, Bennett DA. Total daily physical activity and the risk of AD and cognitive decline in older adults. Neurology 2012; 78(17):1323–1329. doi: 10.1212/WNL.0b013e3182535d35 22517108 10.1212/WNL.0b013e3182535d35 PMC3335448

[49] World Health Organization (WHO). The epidemiology and impact of dementia: current state and future trends. Geneva: World Health Organization; 2012.

[50] Satizabal CI, Beiser AS, Chouraki V, Chêne G, Dufouil C, Seshadri S. Incidence of dementia over three decades in the Framingham heart study. N Engl J Med. 2016; 374(6): 523–532. doi: 10.1056/NEJMoa1504327 26863354 10.1056/NEJMoa1504327 PMC4943081

