# Supplementary material for: An assessment of direct and indirect costs of dementia in Brazil
Source: PLoS One. 2018 Mar 1;13(3):e0193209. doi: 10.1371/journal.pone.0193209 (PMC5832239; doi:10.1371/journal.pone.0193209)
Supplement: S1 Table — (DOCX) [file pone.0193209.s001.docx]

## S1 TABLE. Table 1. Sociodemographic characteristics and clinical status of patients, according to patient FAST category. Brazil, 2016.

| Patient characteristics | | Patient dementia stage | | | |  |
| --- | --- | --- | --- | --- | --- | --- |
|  |  | Mild | Moderate | Severe | Total | Sig. |
| Patients | N | 61 | 74 | 21 | 156 |  |
|  | % | 39.10 | 47.44 | 13.46 | 100.00 |  |
| Sex |  |  |  |  |  |  |
| Male | (%) | 39.34 | 43.24 | 42.86 | 41.67 | # |
| Female | (%) | 60.66 | 56.76 | 57.14 | 58.33 | # |
| Age (years) | Mean | 72.23 | 72.97 | 74.57 | 72.90 | # |
|  | SD | 9.99 | 10.62 | 9.57 | 10.20 |  |
| Retired | (%) | 85.25 | 79.73 | 76.19 | 81.41 | # |
| Income (US$) | Mean | 273.04 | 323.12 | 195.92 | 286.41 | # |
|  | SD | 233.91 | 444.56 | 163.60 | 345.74 |  |
| Diagnosis |  |  |  |  |  |  |
| Alzheimer's disease | (%) | 63.93 | 74.32 | 57.14 | 67.95 | # |
| Vascular dementia | (%) | 4.92 | 0.00 | 0.00 | 1.92 | # |
| Frontotemporal dementia | (%) | 4.92 | 2.70 | 19.05 | 17.10 | # |
| Lewy Body dementia | (%) | 0.00 | 1.35 | 0.00 | 0.64 | # |
| Other | (%) | 26.23 | 21.62 | 23.81 | 23.72 | # |
| Disease evolution (months) | Mean | 51.48 | 60.82 | 82.86 | 60.13 | * |
|  | SD | 39.04 | 39.39 | 47.99 | 41.44 |  |
| Comorbidities (n) | Mean | 2.13 | 2.12 | 1.38 | 2.03 | # |
|  | SD | 1.36 | 1.32 | 1.50 | 1.38 |  |
| Diabetes | (%) | 32.79 | 25.68 | 19.05 | 27.56 | # |
| Hypertension | (%) | 62.30 | 67.57 | 28.57 | 60.26 | * |
| Cerebrovascular disease | (%) | 22.95 | 17.57 | 23.81 | 20.51 | # |
| Cardiovascular disease | (%) | 44.26 | 52.70 | 28.57 | 46.15 | # |
| Other | (%) | 50.82 | 45.95 | 38.10 | 46.79 | # |
| Note: * p≤0.05; # p>0.05. SD = standard deviation. | | | | | | |
